# The effect of age and gender on cognitive and psychomotor abilities measured by computerized series tests: a cross-sectional study

**DOI:** 10.3325/cmj.2020.61.82

**Published:** 2020-04

**Authors:** Ivana Pavlinac Dodig, Dona Krišto, Linda Lušić Kalcina, Renata Pecotić, Maja Valić, Zoran Đogaš

**Affiliations:** 1 Department of Neuroscience and Split Sleep Medicine Center, University of Split School of Medicine, Split, Croatia; 2 Dental Medicine, University of Split School of Medicine, Split, Croatia

## Abstract

**Aim:**

To assess age- and gender-associated differences in cognitive and psychomotor abilities measured by the Complex Reactionmeter Drenovac (CRD-series) tests.

**Methods:**

This cross-sectional study, conducted between 2009 and 2019, enrolled 3420 participants (2012 women) aged from 18 to 88 years. The participants solved three CRD-series chronometric tests: discrimination of the light signal position (CRD311), complex psychomotor coordination (CRD411), and simple arithmetic operations (CRD11). We analyzed total test solving time (TTST), minimum single task solving time (MinT), number of errors, initial dissociation, and start, end, and total ballasts as measures of wasted time in the first half of the test, second half of the test, and total test time, respectively.

**Results:**

Age was positively associated with MinT and TTST in all used tests (*P* < 0.001), while initial dissociation, start ballast, and end ballast significantly increased with age (*P* < 0.001). On the CRD11 test, men had shorter TTST than women (*P* = 0.012), shorter start, end, and total ballasts (*P* < 0.001), and made fewer errors than women (*P* < 0.001). On the CRD311 test, women had shorter start (*P* = 0.002), end, and total ballast (*P* < 0.001) than men. On the CRD411 test, men performed better than women on all variables (*P* < 0.001).

**Conclusion:**

Decreased cognitive and psychomotor abilities measured by the CRD-series tests were associated with advanced age. Men performed better than women on simple arithmetic and complex psychomotor coordination tests, whereas women lost less time on the test of light signal position discrimination.

Gender differences exist in specific aspects of cognitive performance, with men having better spatial and mathematical abilities, and women having better verbal fluency and fine motor skills (1,2). These differences, specifically in spatial and orientation skills, appear very early in life as a result of developmental and environmental factors, and spread across the entire life span. Thus, the gender differences in specific cognitive abilities might be related to prenatal or early postnatal sex hormone exposure (1). Previous research revealed that testosterone suppression does not decrease spatial and orientation abilities, but androgens exposure improves spatial-orientation abilities and deteriorates verbal fluency, suggesting the effect of postnatal hormonal exposure (3-5).

The observed gender differences cannot be explained by the differences in the proportion of gray and white matter or the amount and velocity of blood flow in the brain (6-8). Regarding language tasks, women showed stronger bilateral activation engaging diffuse neural circuits, while men relied on unilateral activation (9). Conversely, in spatial tasks, men showed more bilateral neural activation, supporting the idea of cognitive abilities lateralization (10). The use of complex computing tasks and mental rotation of objects demonstrated that women activate additional cerebral regions bilaterally for more complex problem-solving spatial tasks (11). Furthermore, it seems that men and women use distinct brain areas when solving 3D navigation tasks (12).

Gender differences in cognitive abilities are present from early development until old age (1). In elderly people, aging is associated with decreased inhibition control, working memory, response rate, and performance on various problem-solving tasks (13). The observed effects of aging on cognitive processing abilities might be explained by the global processing speed, which contributes to the effectiveness of solving a wide range of cognitive tasks. However, specific age-related differences in cognitive and psychomotor stability during the testing process remain vague. Additionally, age-related differences in the processing speed might be explained by simultaneous structural and functional aging-related changes in the frontal lobes (13-15). Degenerative changes affecting the brain volume, particularly gray matter, are more pronounced in men during middle and late adulthood than in women (16,17). Also, women have larger corpus callosum and their splenium expands more with advanced age (18). Thus, the stronger bilateral representation of the cognitive functions in women may reduce age-associated cognitive decline.

Gender differences in motor performance are two faceted. Women outperform men on the manual dexterity tests assessing fine motor skills (19,20). However, men achieve better results on the tests assessing manual speed and visual perception (21). A new perspective in the evaluation of age and gender effects on cognitive and psychomotor abilities is offered by the use of ballast outcome measures in chronometric approach. Measuring the deceleration toward the end of the testing as a result of tiredness can be used to assess the stability of cognitive and psychomotor functioning. The ballast outcome measures are unique, precise, and reliable, with the results expressed on the metric scale. This represents an advantage over conventional cognitive tests, whose performance indicators are expressed on the ordinal or interval scale (22). The Complex Reactionmeter Drenovac (CRD)-series of chronometric cognitive tests measures the time needed to solve a specific task and offers insights into the neuropsychological mechanisms that are activated while solving these tasks (22).

The aim of this study was to evaluate age and gender effects on some specific aspects of psychomotor performance that have not been investigated yet. Specifically, the purpose of the current study was to assess the stability of cognitive and psychomotor functioning, expressed as ballasts during the process of CRD-series testing. We hypothesized that elderly people would have reduced cognitive and psychomotor abilities with decreased stability compared with the younger population. Furthermore, we hypothesized that men would have better results on simple arithmetic and complex psychomotor coordination tests, whereas no difference was expected in the light signal position discrimination test.

## Material and methods

### Participants

The cross-sectional study recruited 3420 consecutive respondents: students enrolled in the Basic Neuroscience course at the University of Split School of Medicine (USSM) and the University of Mostar School of Medicine, and individuals from the general population of the Split-Dalmatia County. The students were tested during a practical called Reflexes and Reaction Time, with a 100% response rate. The participants from the general population were either researchers’ friends and family members or the visitors of the Department of the Neuroscience Open Days, which were advertised in the local media. The exclusion criteria were age <18 years and severe mental disability. None of the respondents had any previous experience with CRD tests. The study was approved by the USSM Biomedical Research Ethics Committee and was in compliance with the 1964 Helsinki declaration.

### Methods

This study was conducted at the Department of Neuroscience at the USSM and University of Mostar School of Medicine from 2009 to 2019 by highly trained researchers. The tests were conducted in the morning, and all participants were awake ≥1 hour before the testing. The testing was performed at room temperature in a bright and quiet laboratory, which was at the time used only for CRD testing. Only one researcher was present in the laboratory besides the participant. The instrument panels were placed on a white desk, and the windows in front of the participant were covered with shades. Thus, the conditions in the laboratory prevented distraction by external stimuli. The participant sat on a chair with adjustable seat height to be able to optimally reach the CRD panels. Before testing, he or she was informed about the testing purpose and was given test instructions. When the trial test was completed and the test began, the researcher no longer communicated with the participant.

### Instrument

The tests of the CRD series were standardized for the assessment of the dynamic properties of the central nervous system, including speed, stability, accuracy, endurance, and possible functional disturbances during psychomotor and cognitive reactions (22).

Three tests of the CRD-series were used. The CRD11 test assesses convergent thinking and the ability to construct and solve simple mathematic tasks – summation and subtraction to 20. The arithmetic operation is indicated by two light diodes in the upper corners of the panel, while the numbers are indicated by 12 light diodes in the middle part of the panel (horizontal and vertical to the light). To enter the response, the respondent has to press the key in the bottom row of the panel with the index finger.

The CRD311 test assesses observational abilities, detection, identification, and visual perception. Nine small light diodes at the bottom of the panel emit light in a random sequence. To enter the response, the respondent has to press the key below the emitting diode with the index finger of the dominant hand.

The CRD411 test assesses the complex psychomotor eye-hand-leg coordination. Four light diodes in the middle of the panel (two in the top row, and two in the bottom row) emit a light stimulus. To enter the response, the respondent has to press the appropriate buttons by hands if the stimulus is emitted by the top row diodes, press the pedals on the floor by feet if it is emitted by the bottom row diodes, or simultaneously press the buttons and pedals if it is emitted by more than one diode.

The tests consisted of 35 (CRD11, CRD411) or 60 (CRD311) individual tasks, and the transition to the next task was only possible after the correct response had been given. All participants solved the same sequence of individual tasks. CRD testing was conducted sequentially from the simplest (CRD311) to the most demanding test (CRD11).

Several variables were used as descriptors of speed, reliability, stability, emotional excitement and attention: the best (shortest) single-task solving time (MinT), median time to solve the task (MedT), and total test solving time (TTST). The total ballast (TB, total lost time), start ballast (SB, lost time in the first half of the test), and end ballast (EB, lost time in the second half of the test) were indicators of stability. The ballast was calculated as the sum of the differences between the MinT and all the other reaction times during the test (TB = ΣTi-MinT) (2). Start dissociation (D1) manifested as the prolonged time to solve the first five tasks and indicated emotional excitement and distraction when encountering new content or fear of poor outcome. The total number of errors (NErr) indicated attention and vigilance.

### Statistical analysis

Continuous data are presented as mean ± standard deviation, and age is presented as median (min, max). The normality of the distribution of residuals was tested by the Kolmogorov-Smirnov test. A one-way MANCOVA was performed to test the significance of differences in reaction time test performance (MinT, MedT, NErr, SB, EB, TB) between men and women after controlling for age as a covariate. Therefore, the dependent composite variable in the MANCOVA model for CRD11 and CRD411 included MinT, MedT, TTST, NErr, SB, EB, and TB, while for CRD311 it did not include NErr. The pairwise comparisons of dependent variables (after age adjustment) between men or women were performed with ANCOVA. The effect sizes of differences were reported as partial η^2^. We calculated predicted values of the GLM analysis in order to plot their association with age in both genders. Regression analysis included MinT as a dependent variable, and age, start ballasts, end ballast, and start dissociation as independent variables. All variables were centered, and interaction effects of ballasts and age, and start dissociation and age were tested. Statistical analysis was conducted with MedCalc, version 11.5.1.0 (MedCalc Software, Mariakerke, Belgium) and SPSS, version 14 (IBM, Armonk, NY, USA).

## Results

Of 3420 participants, 2012 (58.8%) were women. The average age of men was 31 (18-88) and of women 22 (18-84) (Table 1). There was a significant adjusted mean difference in the combined dependent variable on CRD11 (Wilks’ λ = 0.961; *P* < 0.001), CRD311 (Wilks’ λ = 0.974; *P* < 0.001), and CRD411 (Wilks’ λ = 0.919; *P* < 0.001) in favor of men (after age adjustment). Also, age had a significant effect on the combined dependent variable on all tests. The gender effect (after age adjustment) was most pronounced on CRD411 test (Wilks’ λ = 0.423, partial η^2^ = 0.577, *P* < 0.001) (Table 2). Women had longer reaction time on all reported measures on CRD11 and CRD411. On CRD11, the largest effect size was reported for TTST and MedT (Δ = 15.996, partial η^2^ = 0.037, *P* < 0.001 and Δ = 2.986, partial η^2^ = 0.031, *P* < 0.001; respectively) (Table 3). On CRD311, the effect sizes of differences were not as high as on other tests. In addition, women had shorter EB and TB than men (partial η^2^ = 0.005, *P* < 0.001, and partial η^2^ = 0.001, *P* = 0.04; respectively) (Table 3). On CRD411, women had longer reaction times than men, which was most pronounced for MinT and MedT (Δ = 0.659, partial η^2^ = 0.060, *P* < 0.001 and Δ = 1.335, partial η^2^ = 0.068, *P* < 0.001; respectively).

**Table 1:** Distribution of participants by age group

	No. (%) of participants		
	total	men	women
Age (years)	3420 (100.0)	1408 (41.2)	2012 (58.8)
18-24	1770 (51.8)	630 (35.6)	1140 (64.4)
25-34	229 (6.7)	109 (47.6)	120 (52.4)
35-44	229 (6.7)	107 (46.7)	122 (53.3)
45-54	350 (10.2)	177 (50.6)	173 (49.4)
55-64	458 (13.4)	202 (44.1)	256 (55.9)
65-74	289 (8.5)	138 (47.8)	151 (52.2)
75 and more	95 (2.8)	45 (47.4)	50 (52.6)

**Table 2:** Main effects of gender and age on the composite measure in each CRD test*^†^

	Wilks’ λ	F	P	Partial η^2^
CRD11				
gender	0.961	25.988	<0.001	0.039
age	0.403	954.910	<0.001	0.597
CRD311				
gender	0.974	22.721	<0.001	0.026
age	0.408	1238.031	<0.001	0.592
CRD411				
gender	0.919	54.207	<0.001	0.081
age	0.423	834.354	<0.001	0.577

*MinT – the best (shortest) single-task solving time, MedT – median time to solve task, TTST – total test solving time, NErr – total number of errors on the test, SB – start ballast, EB – end ballast, TB – total ballast.

†MANCOVA performed including MinT, MedT, TTST, NErr, SB, EB, TB as dependent variables, testing main effects of age and gender.

**Table 3:** Analysis of gender effect including age as a covariate on specific outcomes on CRD tests*^†^

	Adjusted mean difference female-male	SE	F	P	Partial η^2^
CRD11					
MinT	1.445	0.173	69.402	<0.001	0.021
MedT	2.986	0.295	102.159	<0.001	0.031
TTST	15.996	14.405	123.311	<0.001	0.037
NErr	0.692	0.109	40.680	<0.001	0.012
SB	57.785	6.173	87.615	<0.001	0.026
EB	51.608	5.626	84.149	<0.001	0.025
TB	10.939	10.859	101.486	<0.001	0.030
CRD311					
MinT	0.217	0.027	66.039	<0.001	0.019
MedT	0.158	0.038	17.575	<0.001	0.005
TTST	10.742	1.824	34.691	<0.001	0.010
SB	0.021	0.684	<0.001	0.975	<0.001
EB	- 2.309	0.578	15.945	<0.001	0.005
TB	- 2.288	1.113	4.225	0.040	0.001
CRD411					
MinT	0.659	0.047	194.923	<0.001	0.060
MedT	1.335	0.089	223.841	<0.001	0.068
TTST	109.782	7.684	204.106	<0.001	0.062
NErr	2.911	0.399	53.346	<0.001	0.017
SB	37.691	3.151	143.057	<0.001	0.045
EB	49.042	4.364	126.307	<0.001	0.040
TB	86.733	6.879	158.958	<0.001	0.049

*MinT – the best (shortest) single-task solving time, MedT – median time to solve task, TTST – total test solving time, NErr – total number of errors on the test, SB – start ballast, EB – end ballast, TB – total ballast.

†Pairwise comparisons following MANCOVA testing the effect of gender and including age as a covariate on specific outcomes (MinT, MedT, TTST, NErr, SB, EB, TB) on CRD tests, including adjustment for multiple comparisons.

**Figure 1:**
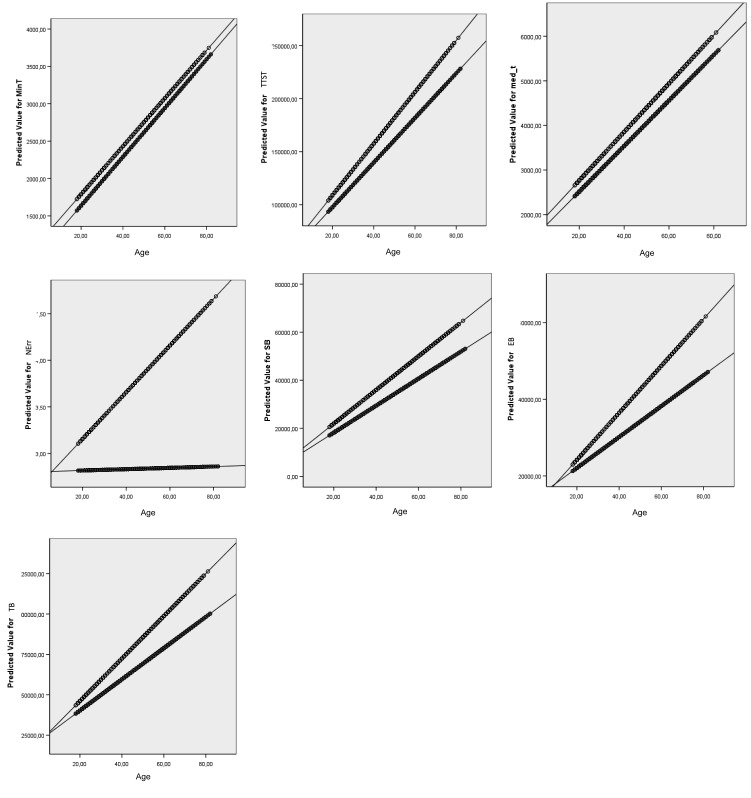
Regression lines of predicted values in each variable of the CRD11 test associated with age in women (gray circles) and men (black circles). MinT – the best (shortest) single-task solving time, MedT – median time to solve task, TTST – total test solving time, NErr – total number of errors on the test, SB – start ballast, EB – end ballast, TB – total ballast.

**Figure 2:**
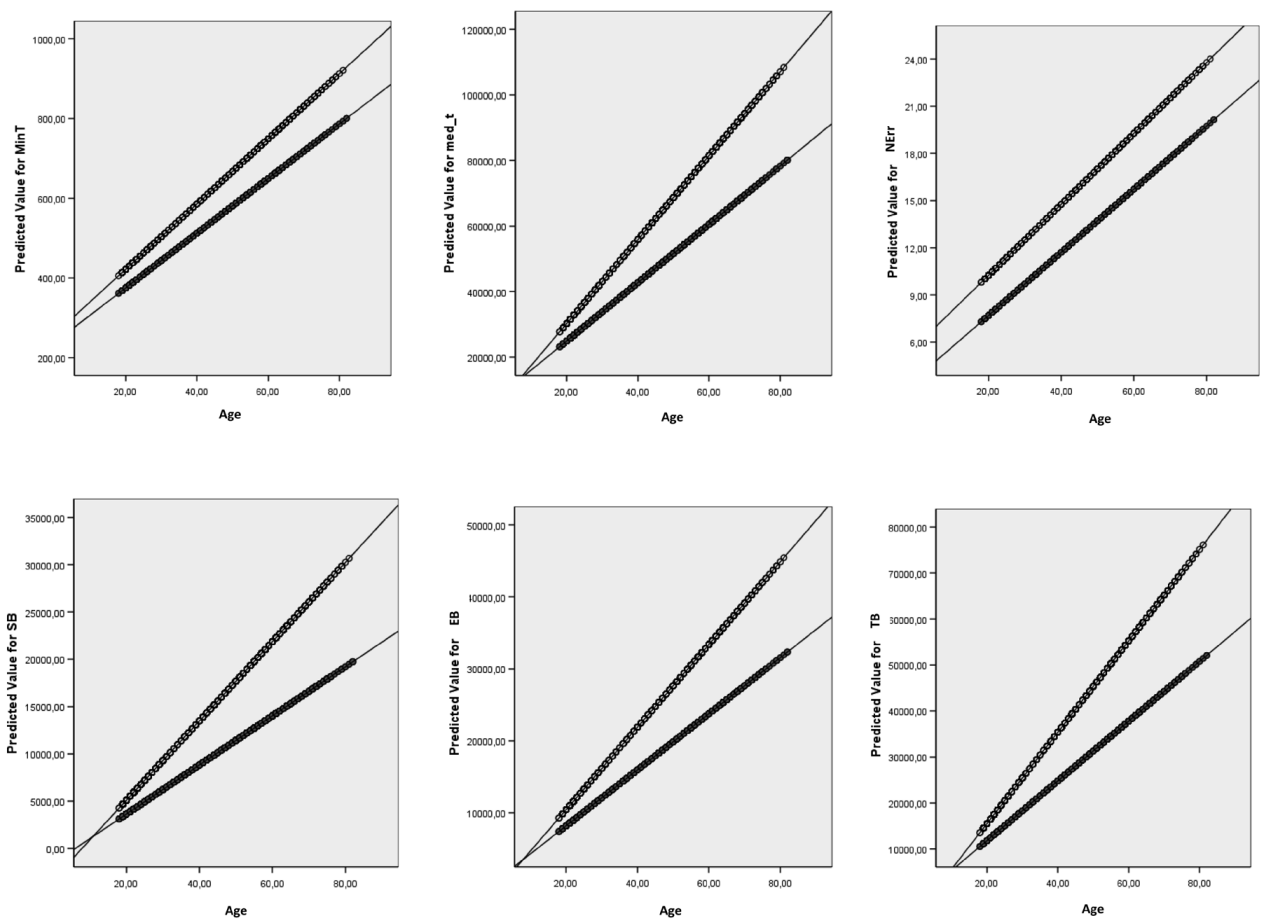
Regression lines of predicted values in each variable of the CRD411 test associated with age in women (gray circles) and men (black circles). MinT – the best (shortest) single-task solving time, MedT – median time to solve task, TTST – total test solving time, NErr – total number of errors on the test, SB – start ballast, EB – end ballast, TB – total ballast.

In order to test the interaction effects, we included the interaction of age and gender in the model (Table 4). A significant interaction effect of age and gender on the composite variable of CRD tests was reported on CRD11 and CRD411. On CRD11, advanced age and female gender were associated with prolonged reaction time (TTST), increased number of errors, and longer ballast times (Table 4, 5, and Figure 1). On CRD411, advanced age and female gender were associated with prolonged reaction times (MinT, MedT, and TTST) and longer ballasts (Table 4, 5, and Figure 2). On CRD311, age and gender did not affect the test results (Table 4 and 5). Women had more pronounced impairment of reaction times with advanced age on CRD11 (Figure 1) and CRD411 (Figure 2).

**Table 4:** The analysis of overall interaction effects of age and gender, followed by effects on specific CRD tests’ outcomes*^†^

	Wilks’ λ	F	P	partial η^2^
CRD11	0.981	12.175	<0.001	0.019
MinT		0.300	0.584	<0.001
MedT		3.858	0.050	0.001
TTST		16.783	<0.001	0.005
NErr		16.130	<0.001	0.005
SB		16.220	<0.001	0.005
EB		44.370	<0.001	0.014
TB		32.955	<0.001	0.010
CRD311	0.999	0.676	0.609	<0.001
MinT		1.069	0.301	<0.001
MedT		2.067	0.151	<0.001
TTST		2.091	0.148	<0.001
SB		0.749	0.387	<0.001
EB		0.449	0.503	<0.001
TB		0.774	0.379	<0.001
CRD411	0.963	23.828	<0.001	0.037
MinT		25.294	<0.001	0.008
MedT		62.972	<0.001	0.0201
TTST		83.003	<0.001	0.026
NErr		1.241	0.265	<0.001
SB		82.419	<0.001	0.026
EB		57.526	<0.001	0.018
TB		80.457	<0.001	0.026

*MinT – the best (shortest) single-task solving time, MedT – median time to solve task, TTST – total test solving time, NErr – total number of errors on the test, SB – start ballast, EB – end ballast, TB – total ballast.

†MANCOVA performed including MinT, MedT, TTST, NErr, SB, EB, TB as dependent variables, testing the interaction effects of age and gender, followed by specific CRD outcomes interaction effects.

**Table 5:** Regression coefficients on each outcome variable of the CRD test regarding main and interaction effects of age and gender*

				Standardized coefficients	t	P
	R	Adjusted R Square	P	Beta		
**CRD11**	0.806	0.648	<0.001			
**Age**				0.569	44.155	<0.001
D1				-0.018	-0.731	0.465
SB				0.209	7.708	<0.001
**E**B				0.161	7.927	<0.001
D1 **× age**				0.061	3.041	0.002
**S**B **× age**				0.049	1.378	0.168
**E**B **× age**				-0.119	-3.932	<0.001
**CRD311**	0.779	0.607	<0.001			
**Age**				0.663	51.460	<0.001
D1				-0.131	-5.346	<0.001
SB				0.291	10.319	<0.001
EB				-0.444	-25.768	<0.001
D1 **×** age				-0.028	-0.938	0.348
SB **×** age				0.017	0.489	0.625
EB **×** age				0.288	14.885	<0.001
						
**CRD411**	0.774	0.598	<0.001			
**Age**				0.477	32.624	<0.001
D1				0.140	3.919	<0.001
SB				0.016	0.332	0.740
EB				0.242	7.681	<0.001
D1 **×** age				-0.037	-1.029	0.304
SB **×** age				0.104	2.284	0.022
EB **×** age				-0.032	-1.051	0.293

*D1 – start dissociation, SB – start ballast, EB – end ballast.

**Figure 3:**
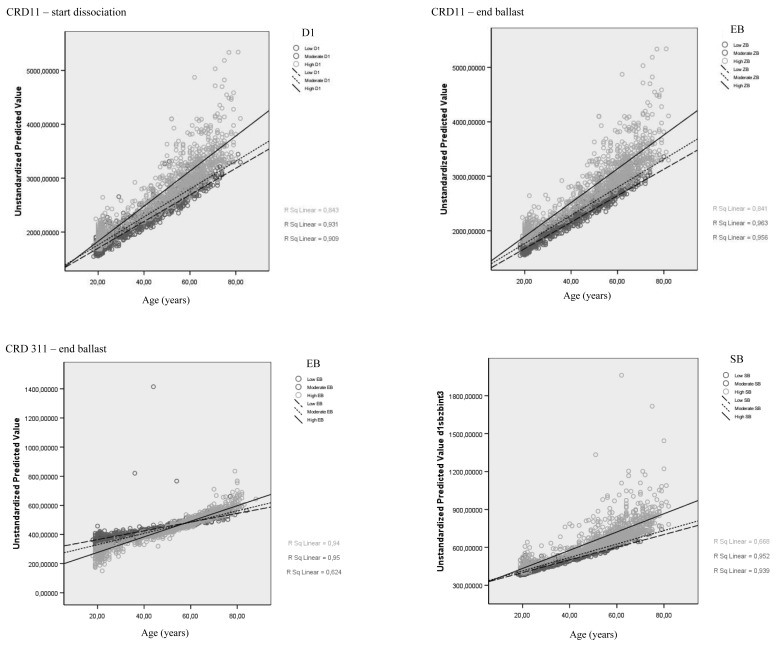
Regression lines of the best (shortest) single-task solving time (MinT) predicted values associated with age in low, moderate, and high start ballast (SB), end ballast (EB), and start dissociation (D1) group.

In order to assess if the contribution of ballasts and start dissociation to MinT reaction time depended on age, a regression analysis was performed, including MinT as a dependent variable and age, SB, EB, and D1 as independent variables. On CRD11, the model explained 64.8% of variance in MinT, and longer D1 and longer EB contributed more to the age-related prolongation of MinT (Figure 3). On CRD411, the model explained 59.8% of variance, and longer SB contributed more to the age-related prolongation of MinT (Figure 3). On CRD311, the model explained 60.7% variance, and longer EB contributed more to age-related prolongation of MinT (Figure 3). Regression coefficients of each outcome variable of three CRD tests regarding main and interaction effects of age and gender are presented in Table 5.

## Discussion

This study showed that elderly participants had reduced cognitive and psychomotor abilities measured by CRD-series tests compared with younger participants. Furthermore, after age adjustment men were shown to achieve better results than women in psychomotor coordination and convergent thinking, while women lost less time than men in the discrimination of light signal position.

Better results in convergent thinking achieved by men are consistent with previous findings of gender differences in mathematical and quantitative tasks (1). However, women were previously shown to have better results when it came to some mathematical abilities, indicating the complicated background of gender-related differences in cognitive abilities (1). Even though gender-related cognitive differences might be explained by different neuroanatomic, biologic, and neurophysiological characteristics of the male and female brain (1,6-8), psychological differences should also be taken into account. Motivation, previous learning, or test-related anxiety all failed to independently explain the differences, and the exact underlying mechanisms remain to be elucidated (1). Better results on psychomotor tasks in men might be the result of the type and style of play during childhood. This, together with their spatial abilities (1,23,24), could explain the observed differences on the CRD11 and CRD411 tests. Visuospatial abilities begin to develop in early childhood, last for a lifetime, and can be advantageous for some aspects of mathematical performance (1).

Our results showed that even though men had shorter MinT and TTST on the test of light signal position discrimination, women were less susceptible to detrimental effect of fatigue. This is contradictory to the results of the previous studies that reported better visuospatial abilities in men. However, we presume that the performance on the CRD311 test might be associated with some other aspects of cognitive processing, as well as with better fine motor activity in women (25). It might be also associated with the fact that among the used tests, CRD311 best correlated with the results of the O'Connor Dexterimeter, which measures the speed and agility of finger and hand movements (unpublished data). Better performance of women on some tests was explained by finger size (26). Therefore, the performance on the light signal position discrimination test might be associated with fine motor skills, as well as with the speed and agility of the small muscles of the hand. Regarding perceptive abilities, women could detect the signal better than men because they have larger visual field, better close-up vision, and slightly different color perception (27,28). Moreover, visuospatial abilities are not a single measure but possess multiple components (1). Gender effect on visuospatial abilities also depends on the task complexity, with the smallest effect size for the simplest and commonly used tasks (1). In our study, the test used to measure visual perception of the light signal position can be considered quite simple compared with other tests assessing visuospatial abilities (such as 3D mental rotation of the objects) and it probably assesses visual perception rather than visuospatial abilities, which include visual orientation and processing location of objects in space (29). This, along with the possible influence of the fine motor skills, might have contributed to our results, indicating the complicated background of gender-related differences in cognitive abilities.

The progressive decrease in cognitive and psychomotor performance associated with advanced age found in our study is not surprising since previous studies showed that aging decreased cognitive abilities (13-15). Age-related deterioration of cognitive and psychomotor abilities was more pronounced in women in the domains of complex psychomotor coordination and convergent thinking, but not in the discrimination of the light signal position. These discrepancies might be the consequence of the specific neuroanatomical substrates of the different neuronal loss in men and in women due to aging (16,17).

This study demonstrated that ballasts affected the deterioration of results associated with advanced age. Indeed, our results demonstrated that on the test of light signal position discrimination and on the test of convergent thinking, age-related deterioration of reaction time was associated with prolonged end ballast. However, on the test of complex psychomotor coordination, it was associated with prolonged start ballast, a finding that is not surprising for a test that requires the simultaneous movement of both hands and feet. Specifically, the elderly population might have reduced motor response, usually owing to pathophysiological age-related processes affecting the joints (30,31).

The limitations of this study include an unequal quantitative distribution of the participants among the age groups. In addition, the youngest adult age group mostly comprised medical students, who are likely to have higher cognitive abilities. The level of education of participants other than students was unknown. However, age and gender have been shown to more strongly affect performance in adults than education (21). Cognitive abilities are usually reduced in individuals who suffer from sleepiness, dehydration, fatigue, or chronic diseases (32-35). To minimize the effect of sleepiness and fatigue, all the tests were performed during weekday mornings. Additionally, no information about possible chronic conditions or medications taken by the participants was available. Old people, who usually take more medications, often have chronic diseases, which might have intensified the cognitive and psychomotor impairment. As our respondents probably had a wide range of diagnoses and received prescribed medications, these confounders presumably had various effects on the cognitive and psychomotor abilities. Although the majority of the observed effects probably would not have been substantially changed if the confounders had been accounted for, still they should be considered highly relevant.

In conclusion, this study showed that decreased cognitive and psychomotor abilities measured by the CRD-series tests were associated with advanced age. Men had better results on simple arithmetic and complex psychomotor coordination tests, whereas women lost less time on the light signal position discrimination test. Furthermore, women showed a stronger association of deteriorating complex psychomotor coordination abilities and convergent thinking with advanced age.
